# Application of hybrid blocking layers in solid-state dye-sensitized solar cells

**DOI:** 10.1186/s40064-015-1140-2

**Published:** 2015-09-17

**Authors:** Philipp Lellig, Michael Meister, Jannis W Ochsmann, Martin A Niedermeier, Monika Rawolle, Frédéric Laquai, Peter Müller-Buschbaum, Jochen S Gutmann

**Affiliations:** Department of Chemistry and Center for Nanointegration Duisburg-Essen (CENIDE), University of Duisburg-Essen, 45141 Essen, Germany; Technische Universität München, Lehrstuhl für Funktionelle Materialien, James-Franck-Straße 1, 85748 Garching, Germany; Max-Planck-Institut für Polymerforschung, Ackermannweg 10, 55128 Mainz, Germany

**Keywords:** Hybrid, Blocking layer, Titanium dioxide, Solar cells

## Abstract

A hybrid blocking layer consisting of a conducting TiO_2_ network embedded in a ceramic matrix is implemented in a solid-state dye-sensitized solar cell. This novel type of blocking layer is thinner than the classical blocking layer films as shown with SEM and XRR measurements, and thereby the conductivity of the hybrid film is increased by 110%. A percolating TiO_2_ network, proven by TEM/ESI and GISAXS measurements, allows for the charge transport. Although being thinner, the hybrid film completely separates the rough electrode material from the hole-transport medium in solar cells to avoid the recombination of charge carriers at this interface. In total, the power conversion efficiency of solar cells is improved: the application in photovoltaics shows that the efficiency of devices with the hybrid blocking layer is increased by 6% compared to identical solar cells employing the conventional blocking layer.

## Background

Hybrid solar cells are cost-effective alternatives to their expensive, silicon-based counterparts (Oregan and Grätzel 1991). In dye-sensitized solar cells (DSSCs) inorganic, n-type metal oxides are combined with solid- or liquid-state hole-transport materials (Kwong et al. 2004; Wonjoo et al. 2008; Gur et al. 2006; Andrew et al. 2006; Cui et al. 2006; Beek et al. 1009; Kudo et al. 2007). In most cases a mesoporous, dye-sensitized titanium dioxide film (TiO_2_) is used as acceptor-material (Yun-Yue et al. 2008). In so-called *Grätzel* cells a TiO_2_ film is combined with a liquid electrolyte yielding power conversion efficiencies beyond 10% (Grätzel 2006). Despite such high power conversion efficiencies, this type of solar cell has not yet established due to problems related to the liquid electrolyte. To avoid leakage of the electrolyte and to increase the lifetime of the devices, the liquid electrolyte can be replaced by organic, solid-state hole-transport materials (Snaith and Grätzel 2006; Schmidt-Mende and Grätzel 2006). Especially for these so-called solid-state DSSCs the use of a blocking layer (BL) is essential for the proper device functionality (Howie et al. 2007; Kavan and Grätzel 1995; Williams et al. 2008; Yoshida et al. 2008). The blocking layer usually consists of a compact TiO_2_ film which is deposited between the transparent conductive oxide (TCO) coated glass and the hole-transport material (HTM). This layer physically separates the transparent front electrode from the HTM. The implementation of this additional layer creates a rectifying contact between the n-type TiO_2_ in the blocking layer and the p-type HTM, so that the charge recombination between electrons and holes at the TCO/HTM-interface is avoided. This way, the photocurrent output is increased significantly and, moreover, it has been found that in some cases solid-state DSSCs show no power conversion efficiency in the absence of the blocking layer and instead a linear current–voltage relation is observed (Peng et al. 2004).

The conventional blocking layer is prepared either by spray pyrolysis or spin coating (Cameron and Peter 2003; Hart et al. 2006; Wu et al. 2010; Grant et al. 2003). The most important prerequisite for the functionality of the blocking layer is to produce a closed film on the rough TCO substrate. However, crack formation of inorganic materials upon drying, sintering and cooling is a common problem in the production of such thin films (Mizuno et al. 1998; Jagota and Hui 1992; Mah et al. 2003). Therefore, a certain thickness of the TiO_2_ film is required to obtain a closed film that is not penetrable by the hole-transport material: the minimum thickness of the conventional blocking layer, as determined by Peng et al. (2004), is 120 nm. If the film thickness is lower, the efficiency drops significantly because of crack formation. However, the series resistance of the blocking layer increases with increasing film thickness. Therefore, a further reduction of the blocking layer film thickness is desirable as this improves the charge carrier transport in solid-state DSSCs.

The reduction of the blocking layer thickness can be achieved by using a hybrid material instead of a pure inorganic film. Recently, the application of novel block copolymers to fabricate hybrid films containing nanostructured TiO_2_ with integrated function was reviewed (Rawolle et al. 2012). Block copolymer-assisted templating is a versatile technique to produce inorganic materials of defined structure, size and shape on large scale areas (Jung and Ross 2009; Niedermeier et al. 2012; Falaras et al. 2008). The self-assembly of diblock copolymers possessing two thermodynamically incompatible polymer chains leads to the formation of 3-dimensional ordered microstructures without external direction. In combination with metal oxide precursors in sol–gel chemistry, the polymer acts as a template and its morphology is transferred to the inorganic material (Rethore and Pandit 2010; Kim et al. 2008). By altering the composition of the sol–gel mixture or by varying the block length ratio of the templating polymer, a variety of different structures is obtained in a convenient way (Cheng et al. 2007). Often the polymer is combusted after being used as templating agent and deals no further purpose. However, if the polymer carries a further functionality this step is not applicable and in this case the polymer is used to fabricate an organic/inorganic hybrid material (Lechmann et al. 2009).

A hybrid blocking layer, which we had developed, is a competitive alternative to the classical approach (Memesa et al. 2006, 2009). In our approach, the amphiphilic diblock copolymer poly(ethylene oxide)-block-poly(dimethylsiloxane)methylmethacrylate (PEO-MA(PDMS)) is used as templating agent together with a TiO_2_ precursor in sol–gel-chemistry. Such a type of amphiphilic diblock copolymer is used to fabricate TiO_2_ nanostructures with a high structural order (Rawolle et al. 2011). Ethylene oxide containing polymers are widely used for structuring titania films, since it is known that metal oxides coordinate to the ethylene oxide, thereby allowing for excellent templating of percolating TiO_2_ networks (Rawolle et al. 2012; Gutierrez et al. 2008; Yang et al. 1998). The hydrophobic poly(dimethylsiloxane) (PDMS) turns into an insulating ceramic material upon calcination in inert atmosphere (Corriu et al. 1997). In the calcination step the polymer acts as a crosslinking agent and fills up voids in the TiO_2_ network to yield a closed film—even at a low film thickness. The result is a very thin and crack-free hybrid film that contains a crystalline, conducting TiO_2_ network.

Recently, we were able to show that the implementation of the hybrid blocking layer in solid-state DSSCs is feasible: (Lellig et al. 2012). Electrical conductivity through the hybrid blocking layer was validated by conductive scanning probe microscopy. The direct comparison of the novel hybrid blocking layer and the conventional compact TiO_2_ film revealed that the conductivity of the hybrid blocking layer increased by 32%. The functionality of the hybrid blocking layer, i.e. the prevention of short-circuits, was verified by the implementation in solid-state DSSCs. In doing so, the efficiency of the device with the hybrid blocking layer was increased from 0.35 to 1.27% compared to an identical device omitting the blocking layer.

However, the efficiency of solid-state DCCSs with a conventional blocking layer was not reached due to insufficient charge transport between the hybrid blocking layer and the mesoporous TiO_2_ film in the photoactive layer. In the present investigation, the molecular weight ratio of the templating agent is altered to improve the distribution of the titanium dioxide particles within the hybrid material to encounter this issue and increase efficiencies. For this purpose, another batch of the amphiphilic diblock copolymer PEO-MA(PDMS) has been synthesized with a PEO to PDMS molecular weight ratio of ~1:1. Furthermore, the spin coated films have been treated by solvent vapor annealing. Solvent vapor annealing is a common way to enhance the microstructure of solution-casted films (Kim et al. 2004; Fukunaga et al. 2000, 2002; Albert and Epps 2010; Xuan et al. 2004). During the spin coating process the hybrid film is created under high stress. In the vapor annealing step, the spin coated film is swollen by exposure to a solvent vapor atmosphere of tetrahydrofuran (THF) and slowly dried afterwards. By this means, the structure of the film can rearrange. Such improved treatment enhances the conductivity of the hybrid films by 5%. With respect to all additional steps, the films have been prepared in the same way as reported earlier (Lellig et al. 2012). The sol–gel composition with the highest amount of titanium dioxide, resulting in blocking layer films containing 80 wt% TiO_2_ and 20 wt% of the polymer-derived ceramic material, yields the best results in the application in solar cells, and thus we focus on this composition.

In the present investigation, the *conventional* blocking layer, prepared according to the literature, is used as a reference (Yu et al. 2009). The film thickness of the blocking layers is determined by SEM as well as by XRR. The presence of a percolating TiO_2_ network within the hybrid film is verified by GISAXS and TEM/ESI measurements. Furthermore, the electrical conductivity of both types of blocking layer is compared. Finally, the impact of the hybrid blocking layer on the power conversion efficiency is investigated by implementing both types of blocking layers in solid-state DSSCs under identical conditions.

## Results and discussion

### Film thickness

**Figure 1:**
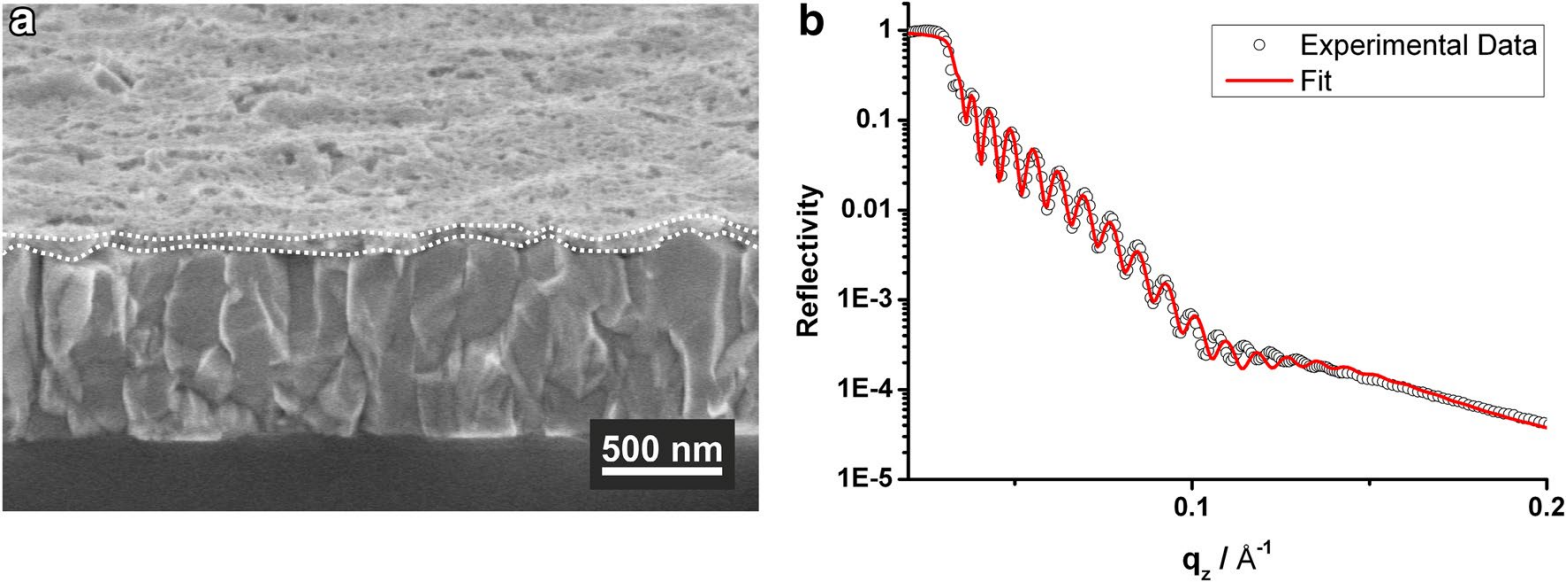
SEM cross section image of the hybrid blocking layer on FTO (the boundaries are indicated by the *dotted line*) (**a**) and XRR data (*circles*) with fit (*line*) (**b**).

The film thickness of the hybrid blocking layer is determined from cross-section scanning electron microscopy (SEM) images and X-ray reflectivity (XRR) measurements as shown in Figure 1. For these measurements, the samples have been prepared on fluorine-doped tin oxide (FTO) and on flat silicon substrates, respectively. The SEM image of the hybrid blocking layer reveals pores on the surface of the film. However, because the templating polymer diffuses into the pores of the TiO_2_ network and turns into a ceramic layer in the calcination step, a closed film is obtained which separates the bottom FTO electrode from the hole-transport layer: the application in solid-state DCCSs in “Current–voltage characteristics” confirms that the hybrid blocking layer is not penetrated by the HTM, as the charge recombination is reduced significantly compared to the device omitting the blocking layer.

The film thickness determined from the SEM image varies locally between 30 and 90 nm due to the high roughness of the FTO. The XRR profile of the hybrid blocking layer shows a superposition of two different layers, i.e. the porous TiO_2_ network and the ceramic material. The fit to the data yields a thickness of (78 ± 2) nm. For the conventional blocking layer, which is used as a reference, a film thickness of (121 ± 2) nm is determined by XRR (data not shown here), representing the optimum thickness for this type of blocking layer. Thus, by using the hybrid material, a reduction of the blocking layer film thickness of 36% is realized. This reduction in film thickness offers the possibility to increase the conductance of the hybrid blocking layer while maintaining its rectifying function.

### Film structure: TEM/ESI

**Figure 2:**
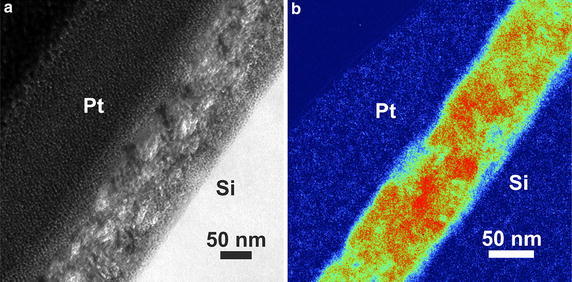
Lamella of the hybrid blocking layer between Pt and Si prepared with a FIB: TEM image (**a**) and TiO_2_ mapping by ESI (**b**). In the ESI image TiO_2_-rich areas are colored *red*, areas of medium TiO_2_-concentration are colored *green* and areas without TiO_2_ are colored *blue.*

The structure of the hybrid film has been investigated by transmission electron microscopy (TEM) and electron spectroscopic imaging (ESI) of a thin lamella of the hybrid blocking layer. For this purpose, a lamella with a diameter below 100 nm has been prepared on a silicon wafer using a focused ion beam (FIB). To increase the mechanical stability of the sample, a layer of platinum (100 nm) was deposited on top of the film. The lamella has been investigated by TEM as well as by ESI to map the distribution of TiO_2_ within the film. The TEM/ESI images are shown in Figure 2.

In the TEM image areas of high electron density, i.e. TiO_2_, appear brighter than the organic material, Pt is black and Si white. Thus, the bright, sphere-like domains in the thin lamella correspond to agglomerates of TiO_2_ particles. These agglomerates are surrounded by the insulating organic material which is visualized by the darker regions. The size of the TiO_2_ domains is ~30 nm and these agglomerates are evenly distributed throughout the layer. These findings are confirmed by the TiO_2_ mapping by ESI: the red, TiO_2_-rich areas match with the bright TiO_2_ agglomerates in the TEM image. The TiO_2_ domains are spread out homogeneously in the hybrid film and they are connected to each other. This leads to the formation of a percolating TiO_2_ network enabling the current flow through the film. Moreover, the TEM and ESI measurements show that, though a medium concentration of TiO_2_ is found at the boundaries of the film, the TiO_2_ particles are well accessible at the surface to allow the charge injection from the photoactive TiO_2_ layer to the blocking layer in solar cells.

### Film structure: GISAXS

**Figure 3:**
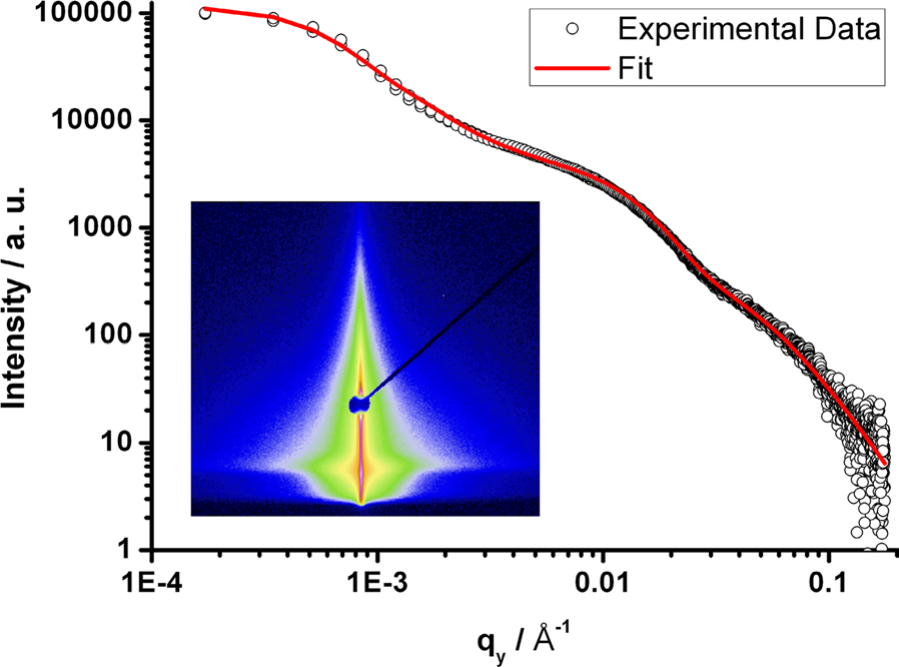
*Horizontal line* cut from 2D GISAXS data, taken at the critical angle of the hybrid layer (*circles*) with fit (*line*). The *inset* shows the 2D GISAXS image.

To investigate the inner 3D structure of the hybrid blocking layer, the hybrid film has been characterized with grazing incidence small angle X-ray scattering (GISAXS) (Müller-Buschbaum 2003). In contrast to microscopy techniques like SEM or TEM which are restricted to probing just a micrometer area, GISAXS offers the possibility to obtain information on the inner structure of the film averaged over a macroscopic area (~1 mm^2^) with nanometer scale resolution. For the GISAXS measurement an incident angle of α_i_ = 0.7° was chosen. This angle is well above the critical angle of TiO_2_ (α_c_ = 0.25°) to ensure that the layer is completely penetrated by the X-ray beam and the structure of the buried TiO_2_ network is investigated. The 2D scattering image of the measurement is shown in the inset of Figure 3.

A horizontal cut (with respect to the sample surface) of the 2D GISAXS data results in a scattering curve which contains information on the lateral structures in the film. For simplicity of the analysis the scattering curve is fitted according to the Unified Fit Model, dividing the cut into linear Porod and shoulder-like Guinier regimes (Lenz et al. 2010). The model describes several structural levels ranging from small particles to larger agglomerates. Thus, characteristic values for the radius of gyration (R_g_) and the fractal dimension (P) of the respective levels are retrieved. The fractal dimension P yields information on the spatial arrangement of the scattering centers.

In the first level, corresponding to the high q region, the fit reveals a radius of gyration of R_g_ = 8 nm and a fractal dimension of P = 3.8 describing a sphere-like morphology. This structure size and shape corresponds to the titanium dioxide crystallites that build up the TiO_2_ network. In the next level, a superstructure with a radius of gyration of R_g_ = 32 nm as well as a fractal dimension of P = 2.5 is found. It corresponds to agglomerates of TiO_2_ crystallites arranged in a 3D order. The radius of gyration of R_g_ = 32 nm is in good agreement with the TiO_2_ domain size of ~30 nm found in the TEM and ESI analysis and the presence of a percolating TiO_2_ network that was already observed in these measurements is confirmed by GISAXS. Due to the GISAXS results, we verified that this percolating network exists on the macroscopic scale. In conclusion, the TiO_2_ particles within the hybrid material build up a macroscopic percolating network which is a prerequisite for the charge carrier transport throughout the film.

### Macroscopic conductance

**Figure 4:**
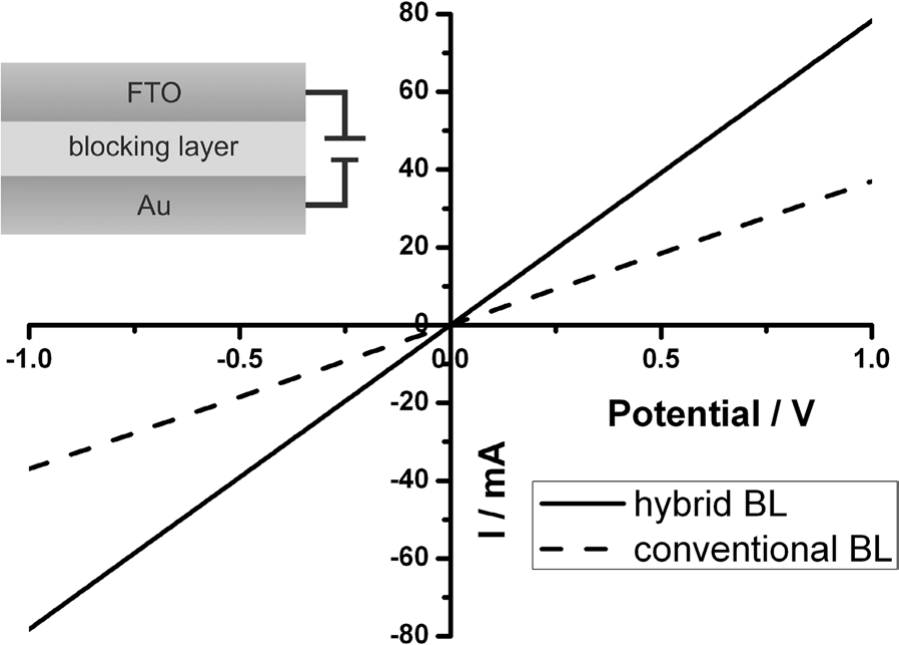
IV-characteristics of the hybrid and of the conventional blocking layer for samples of the structure FTO/blocking layer/Au.

The macroscopic conductance of the hybrid as well as of the conventional blocking layer has been investigated with samples of the structure FTO/blocking layer/Au (see inset of Figure 4). The blocking layer films were prepared on FTO-coated glass and gold counter electrodes with a thickness of 100 nm were deposited by evaporation on top of the films using a shadow mask. This sample structure is identical to the solar cells built in this study, where the photoactive layer, i.e. the dye-sensitized TiO_2_ layer and the hole-transport material, is omitted completely. This way, the current flow through the blocking layers is measured in the same way as in the actual solar cells. As both blocking layers have different thicknesses and the conducting TiO_2_ layers possess different porosities, the conductance rather than the specific conductivity of the materials is measured. A voltage sweep between −1 V and +1 V is carried out and the current flow is recorded. The resulting current–voltage (IV) curves are shown in Figure 4.

For both types of blocking layer a linear current–voltage relation is found, indicating that an ohmic contact is present. The difference in work function of TiO_2_ (Φ = 4.2 eV) and FTO (Φ = 4.3–4.4 eV) is too small to create a rectifying Schottky-contact to result in a diode-like IV-curve (Peng et al. 2004; Qiao and McLeskey 2005). However, these measurements clearly show that the current flow is significantly higher for the hybrid blocking layer than for the conventional blocking layer. The conductance, as determined by the slopes of the curves, is 78 mS for the hybrid and 37 mS for the conventional blocking layer. Thus, the current flow through the hybrid blocking layer is increased by 110%. This increased current flow is attributed to the reduced film thickness and the associated decreased serial resistance of the hybrid blocking layer. Furthermore, it verifies the findings from TEM, ESI and GISAXS, where a percolating TiO_2_ network was found, as the increased current flow shows that no or only few discontinuities are present in the hybrid blocking layer. The increased macroscopic conductance of the hybrid blocking layer promises a positive impact on the power conversion efficiency of solar cells. This impact is investigated in the following section.

### Current–voltage characteristics

**Figure 5:**
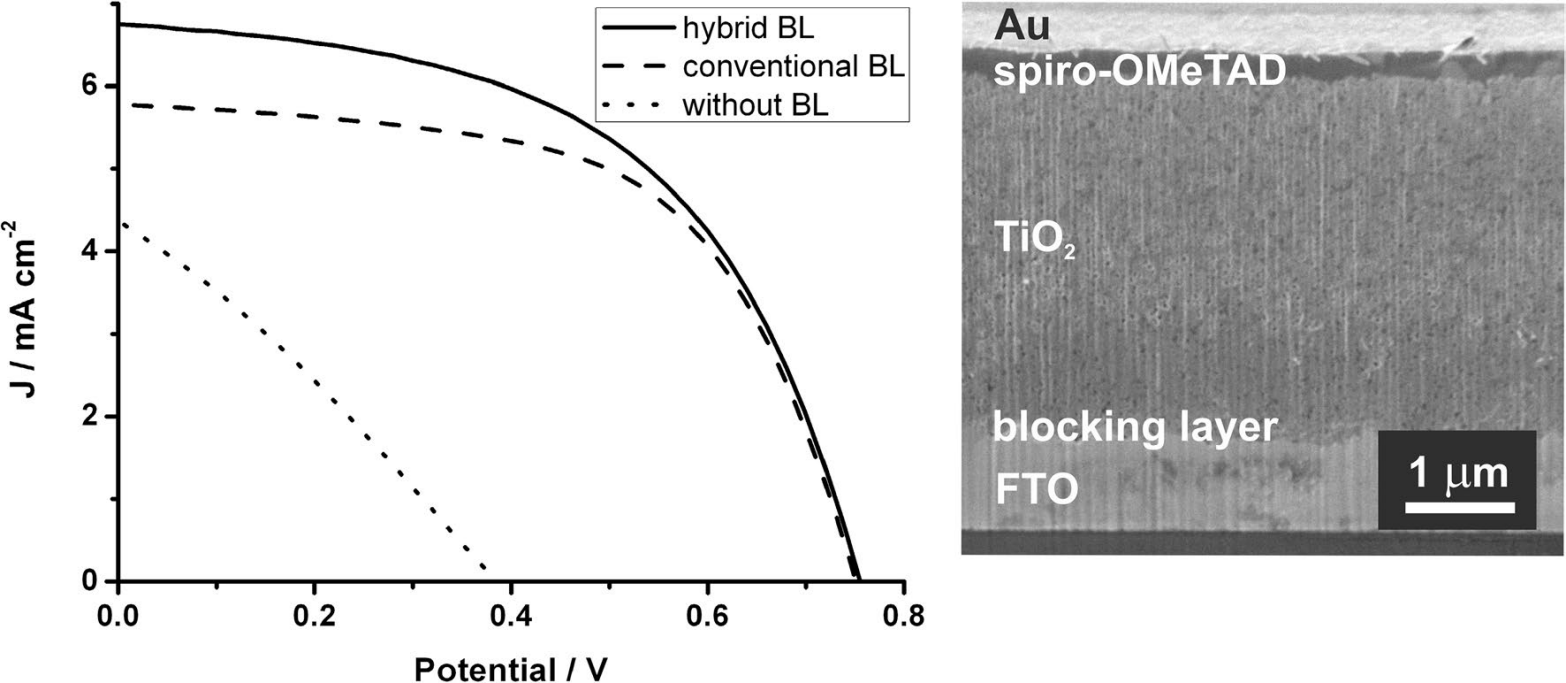
*JV*-curves (*left*) of solar cells with the hybrid (*solid line*), the conventional (*dashed line*) and without blocking layer (*dotted line*) and SEM cross section image (*right*) of a DSSC.

For device characterization the hybrid blocking layer is put into direct comparison to the conventional one by implementing each type of blocking layer in otherwise identical solar cells. Another device without any kind of blocking layer serves as a further reference. Similar to the samples used for the conductivity measurements, the different blocking layers have been prepared on FTO-coated glass. However, a dye-sensitized TiO_2_ layer as well as the hole-transport material 2,2′,7,7′-tetrakis-(*N*,*N*-di-4-methoxyphenylamino)-9,9′-spiro-bifluorene (spiro-OMeTAD) were deposited prior to the evaporation of 6 Au counter electrodes to fabricate functional solar cells. A cross section of a solar cell is depicted in Figure 5, showing the different functional layers. All samples have been assembled in one batch and have been treated exactly the same way in order to relate the differences in the device characteristics to the respective type of blocking layer exclusively. The current density–voltage (JV) curves and their characteristics, averaged over 6 electrodes per solar cell, are shown in Figure 5 and Table 1.

**Table 1:** Characteristics of *JV*-curves

Type of BL	*η*/%	*J*_SC_/mA cm^−2^	*V*_OC_/V	FF/%
Hybrid BL	2.71 ± 0.05	6.75 ± 0.03	0.76 ± 0.01	53 ± 1
Conventional BL	2.56 ± 0.05	5.78 ± 0.03	0.75 ± 0.01	59 ± 1
Without BL	0.50 ± 0.01	4.37 ± 0.02	0.39 ± 0.01	29 ± 0

The solar cell with the hybrid blocking layer exhibits the highest power conversion efficiency with 2.71% followed by the device employing the conventional blocking layer with 2.56%. The reference device without blocking layer is poorly functional which is indicated not only by the low efficiency of 0.50% but also by significantly lower values for the short-circuit current, the open-circuit voltage as well as for the fill factor. Furthermore, the absence of a blocking layer leads to a linear current–voltage relation for this device and demonstrates the necessity of the blocking layer for this type of solid-state DSSCs.

The short-circuit current is 6.75 mA/cm^2^ for the solar cell with the hybrid blocking layer and 5.78 mA/cm^2^ for the solar cell using the conventional blocking layer, thus, an improvement in the short-circuit current of 17% is found. It is noteworthy that the difference in efficiencies between these solar cell is mainly attributed to that difference in short-circuit current, while the values for the open-circuit voltage and fill factor remain basically unchanged. This is a direct evidence for the functionality of the blocking layers: as all devices are identical in construction and under illumination the same amount of charge carriers is generated, the increased short-circuit current shows that the charge recombination is effectively avoided by both types of blocking layers. However, the increased conductivity of the hybrid blocking layer improves the charge transport towards the electrode to increase the extracted photocurrent for this solar cell. Thereby, the efficiency of the solar cells with the hybrid blocking layer is improved by 6% compared to the device employing the conventional blocking layer.

## Conclusions

In summary, we have demonstrated that a hybrid blocking layer, consisting of a TiO_2_ network which is embedded in an insulating ceramic material, is effectively used to avoid charge recombination in solid-state DSSCs. While the percolating TiO_2_ network within the hybrid film allows for current flow, the ceramic material ensures that a closed film on the rough TCO substrate is obtained. By this technique, the hybrid blocking layer is produced much thinner than the conventional, compact layer. Thereby, the hybrid films exhibit a higher conductivity while maintaining their rectifying function to avoid charge recombination at the FTO/HTM interface. The increased conductivity of the hybrid film leads to an increased short-circuit current by 17% in the application in solar cells. This increased conductivity results in an increase in efficiency by 6% compared to solar cells with conventional blocking layer, rendering the hybrid blocking layer a promising alternative for the classical approach.

## Methods

### Diblock copolymer synthesis

PEO-MA(PDMS) diblock copolymer was synthesized by atom transfer radical polymerization of end-functionalized PEO and the macro monomer poly(dimethylsiloxane)methylmethacrylate as described previously (Lellig et al. 2012). However, to obtain a PEO to PDMS molecular weight ratio of ~1:1, 4.27 mL macro monomer was reacted with 2 g of the macro starter. The product was obtained with a number average molecular weight of 14.000 g/mol with a polydispersity of 1.46 as determined by GPC.

### Blocking layer film preparation and solar cell assembly

For the preparation of the hybrid blocking layer we followed the approach developed by Memesa et al., whereat the newly synthesized diblock copolymer PEO-MA(PDMS) was used as templating agent (Rawolle et al. 2012). Furthermore, the plasma etching step was replaced by a solvent vapor annealing treatment. For this purpose, the spin coated films were stored in a desiccator containing a reservoir of THF for 90 min and subsequently dried at room temperature prior to the calcination step.

The conventional TiO_2_-blocking layer was prepared following the method by Yu et al. (2009).

The assembly of the solid-state dye-sensitized solar cells is described elsewhere (Lellig et al. 2012).

### SEM

For the cross sectional analysis, the hybrid blocking layer was prepared on FTO-coated glass and the sample was frozen in liquid nitrogen to avoid delamination of the film upon fracture. The SEM cross-section images were recorded on a LEO 1530 Gemini, Zeiss, with an acceleration voltage of 1–2 kV.

### XRR

XRR measurements of the blocking layer films on flat silicon substrates were conducted on a XRD 3003 TT, Seifert Ltd. GB diffraction system. The collimated X-ray beam was obtained from a Cu anode with a wavelength of λ = 0.l54 nm. The obtained XRR curves were fitted according to the Parratt algorithm using the software Parratt32 (Parratt 1954).

### TEM/ESI

A protective platinum layer (~100 nm) was deposited on top of the hybrid blocking layer prior to the treatment with a focused ion beam (FEI Nova 600). Gallium ions with an energy of 30 keV were used to cut the lamella and reduce its diameter below 100 nm. TEM imaging and a titanium dioxide mapping by ESI of the lamella were conducted on a Tecnai F20, FEI, with an acceleration voltage of 200 kV.

### GISAXS

GISAXS measurements were conducted at the beamline BW4, HASYLAB at DESY, Hamburg, Germany. A beam with a wavelength of λ = 0.138 nm and a beam size of 32 × 17 μm^2^ (horizontal × vertical) was used. For the measurements an incident angle of α_i_ = 0.7° was chosen. The data was fitted according to the Unified Fit Model. Further details can be found in literature (Lenz et al. 2010).

### Conductivity measurements/characterization of solar cells

A Keithley 2400 digital sourcemeter was used to record current–voltage curves for the conductivity measurements as well as for the characterization of the solid-state DSSCs under illumination. The solar cells were characterized under standardized illumination (100 mW/cm^2^, AM 1.5 G standard solar spectrum) using a xenon short-arc lamp, LOT-Oriel. Each sample was measured at 6 different electrodes (area = 0.14 cm^2^) and the averaged values are shown. The deviation of the characteristic values is a result of the temporal instability of the xenon lamp.
